# Defining the oxysterolome: analytical coverage, metabolic connectivity, and biological validation

**DOI:** 10.1016/j.jlr.2026.101170

**Published:** 2026-10-12

**Authors:** Silia Ayadi, Julio Buñay, Chloé Gressein, Sandrine Silvente-Poirot, Marc Poirot, Philippe de Medina

**Affiliations:** Team INOV: "Cholesterol Metabolism and Therapeutic Innovations", Cancer Research Center of Toulouse (CRCT), Inserm, CNRS, University of Toulouse, Toulouse, France

**Keywords:** oxysterolome, oxysterols, cholesterol metabolism, lipid signaling, iterative metabolism, oxysterolomics

## Abstract

The number of oxygenated derivatives of cholesterol, its precursors, and other biologically relevant sterols that exist in biological systems is unknown and may extend substantially beyond the oxysterols currently characterized. Successive oxidation, reduction, hydrolysis, sulfation, glucuronidation, esterification, and conjugation reactions can generate a large chemical space that we refer to as the oxysterolome. However, chemical possibility does not establish endogenous existence, and endogenous existence does not establish biological function. Defining this metabolic space requires a progression from analytical annotation to structural authentication, metabolic reconstruction, and biological validation. Iterative metabolism broadens physicochemical diversity, making analytical coverage method-dependent and requiring complementary approaches for different metabolite classes. Authenticated metabolites should subsequently be connected through precursor–product relationships, enzyme dependence, isotope tracing, metabolic flux, and spatial organization. Biological relevance requires separate evidence linking endogenous exposure and pathway perturbation to molecular mechanisms and phenotypes. Further metabolism can generate, terminate, or redirect biological activity, but these outcomes cannot be predicted from structural complexity. The oxysterolome should therefore be approached not as an expanding catalog of oxygenated sterols, but as an experimentally reconstructed metabolic network integrating molecular identity, pathway connectivity, flux, and biological causality.

## Graphical abstract

**Figure:**
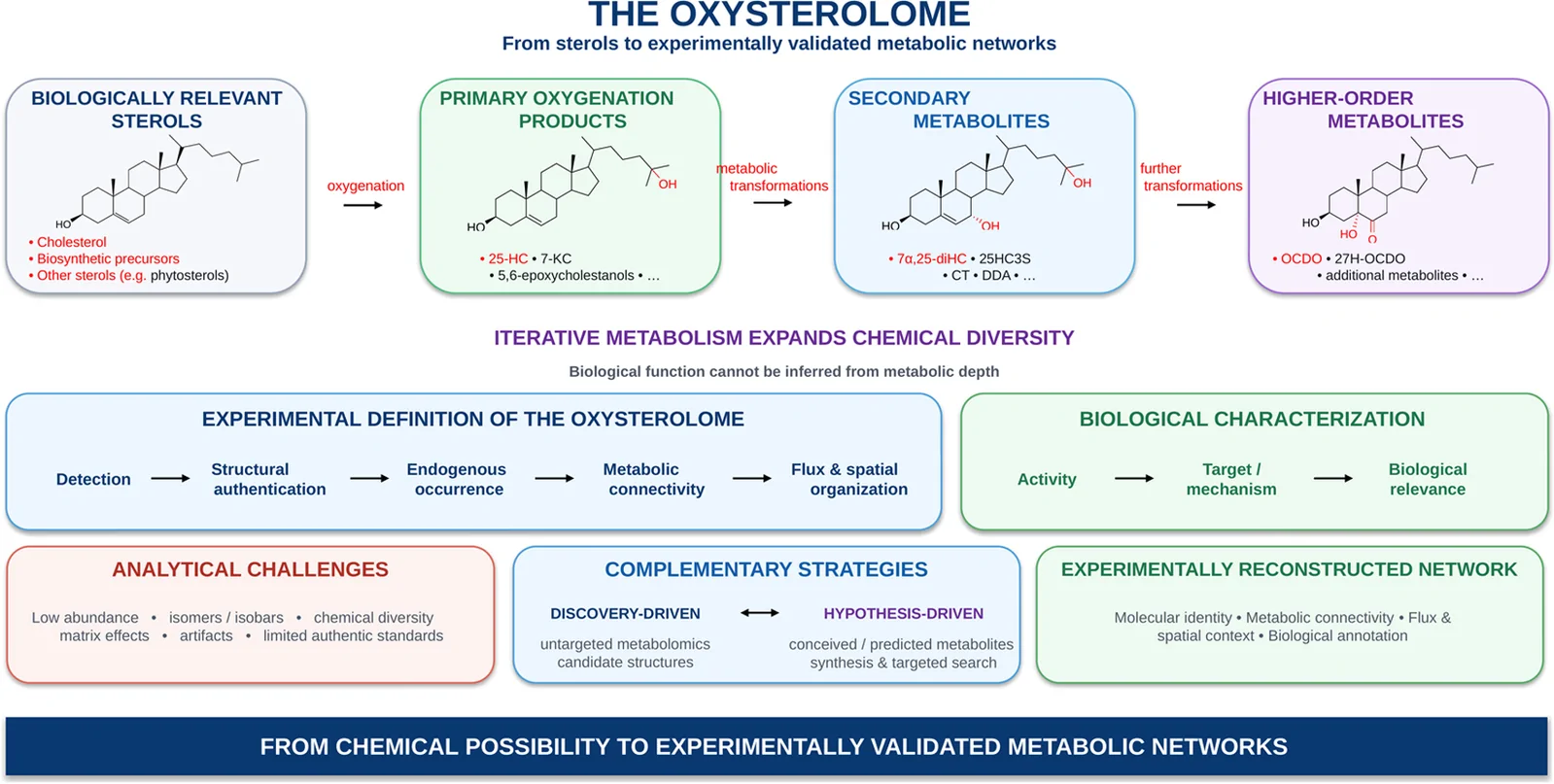

## From oxysterol metabolism to the oxysterolome

Oxysterols are oxygenated derivatives of cholesterol and related sterols generated through enzymatic reactions or nonenzymatic oxidation. In this Review, we use the term oxysterol in a broad biochemical sense to include oxygenation products of cholesterol, cholesterol biosynthetic precursors, and other sterols entering mammalian metabolism, including phytosterols. The oxysterolome further includes products generated through subsequent metabolism of these oxygenated sterols. This operational definition does not imply inclusion of all steroid-derived end products, such as canonical steroid hormones or bile acids, which constitute established metabolic families in their own right, although their pathways may intersect with the oxysterolome. The biological significance of oxysterols has progressively expanded beyond their classical roles as intermediates of sterol and bile acid metabolism or as products of oxidative processes. Individual oxysterols can act through nuclear receptors, G protein-coupled receptors, ion channels, and other molecular targets, although biological activity cannot be generalized to oxysterols as a chemical class. For example, 7α,25-dihydroxycholesterol (7α,25-diHC) is a high-affinity endogenous agonist of EBI2/GPR183 that controls immune-cell positioning, whereas other oxygenated sterols may primarily function as metabolic intermediates or products of transport and elimination 1, 2.

The chemical and metabolic diversity of oxygenated sterols has long been recognized. Early biochemical studies established that cholesterol oxidation generates numerous products and that several of these compounds undergo further enzymatic transformations. Leland Smith’s systematic analysis of cholesterol autoxidation already emphasized both the large chemical diversity of oxygenated cholesterol derivatives and the need to distinguish compounds formed biologically from products generated during isolation or analysis (3). Georges Schroepfer subsequently integrated oxysterol formation, occurrence, metabolism, and biological activity into a comprehensive view of oxysterol biology, while emphasizing the analytical rigor required for structural identification and quantification (4). Thus, the existence of extensive downstream oxysterol metabolism is itself well established and predates the oxysterolome concept.

This metabolic complexity was also revealed by the development of increasingly sophisticated chromatographic and mass-spectrometric approaches. Work by Jan Sjövall *et al*. demonstrated the remarkable diversity of steroids, bile acids, hydroxycholesterols, and their conjugates in biological samples and ultimately contributed to the concepts of “steroidomics” and “cholanoidomics” (5). These studies established an important analytical principle that remains central to the oxysterolome: biological sterol metabolism cannot be understood solely from measurements of a restricted set of metabolites, because hydroxylation and other oxidative transformations can be accompanied by sulfation, glucuronidation, and additional conjugation reactions.

The metabolism of 24(S)-hydroxycholesterol (24(S)-HC) provides a particularly informative historical example. 24(S)-HC is produced predominantly in the brain and undergoes net export into the circulation, thereby providing a route for elimination of cholesterol-derived carbon from the central nervous system 6, 7. Importantly, circulating 24(S)-HC does not represent a metabolic endpoint. Meng *et al*. identified 24(S)- hydroxycholesterol 3-sulfate, 24-glucuronide in serum and urine from children with severe cholestatic liver disease, together with double conjugates of 27-hydroxycholesterol and other oxygenated sterols, demonstrating that successive sulfation and glucuronidation can generate additional levels of sterol diversity (8). Subsequent studies using labeled 24(S)-HC in human hepatocytes and in vivo demonstrated its further conversion into more highly oxygenated sterols and bile acids (9). Together, these observations provide direct evidence that a primary oxysterol can enter multiple successive metabolic transformations involving both further oxidation and conjugation.

These historical developments provide essential foundations for the oxysterolome concept while also defining what remains to be established. The existence of secondary oxysterol metabolism, the occurrence of numerous oxygenated sterols, and the analytical description of complex sterol profiles are not new. Likewise, extensive metabolic transformation is already fundamental to bile acid biology, where successive modifications of the cholesterol scaffold generate interconnected families of metabolites (10). More recently, cholesterol biosynthesis itself has been reconsidered as a dynamic network in which redistribution of metabolic flux changes the availability of sterol intermediates and thereby modifies biological outputs (11). These observations argue against treating individual sterol concentrations independently of the pathways that generate and consume them.

What remains incompletely defined is the extent, connectivity, and biological organization of the metabolic space generated when this principle is applied systematically to oxygenated sterols. A primary oxysterol can undergo additional hydroxylation, oxidation, reduction, epoxide hydrolysis, sulfation, glucuronidation, esterification, or other conjugation reactions. Its products may themselves become substrates for additional transformations. Some of these reactions may primarily promote transport, compartmentalization, turnover, or elimination, whereas others generate metabolites with molecular targets and biological properties distinct from those of their precursors. At present, neither the dimensions of this endogenous chemical space nor the fraction of it associated with biological functions is known.

We use the term oxysterolome to describe the metabolic space formed by oxygenated derivatives of cholesterol, its biosynthetic precursors, and other biologically encountered sterols, together with products generated through their iterative metabolism. The concept therefore builds on, rather than replaces, the extensive biochemical and analytical foundations established by earlier studies of oxysterol and bile acid metabolism. Its purpose is not to rename the collection of known oxysterols or to imply that every theoretically possible oxygenated sterol exists in vivo. Instead, it provides a framework for asking how oxygenated sterols are connected through successive metabolic transformations and how much of the resulting chemical space is actually realized in biological systems. The nomenclature, chemical identity, and metabolic position of representative metabolites discussed throughout this Review are summarized in Table 1, with additional information on their historical characterization and metabolic assignment provided in Supplementary Table S1.

**Table 1:** Nomenclature, chemical identity, and metabolic position of representative metabolites within the oxysterolome

Metabolite name used in this Review	Abbreviation	Historical/common name(s)	Systematic/structural name	Metabolic position^a^	LIPID MAPS ID	PubChem CID
7α-Hydroxycholesterol	7α-HC	7α-Hydroxycholesterol	cholest-5-en-3β,7α-diol	Primary	LMST01010013	107722
24 (S)-Hydroxycholesterol	24 (S)-HC	24S-Hydroxycholesterol; cerebrosterol	cholest-5-en-3β,24S-diol	Primary	LMST01010019	121948
25-Hydroxycholesterol	25-HC	25-OHC	cholest-5-en-3β,25-diol	Primary	LMST01010018	65094
27-Hydroxycholesterol^b^	27-HC	27-Hydroxycholesterol; (25R)-26-hydroxycholesterol	(25R)-cholest-5-en-3β,26-diol	Primary	LMST01010088	123976
7-Ketocholesterol	7-KC	7-Oxocholesterol	7-oxo-cholest-5-en-3β-ol	Primary	LMST01010049	91474
5,6α-Epoxycholestanol^c^	5,6α-EC	5,6α-Epoxycholesterol; cholesterol α-oxide	5α,6α-epoxy-5α-cholestan-3β-ol	Primary	LMST01010011	227037
5,6β-Epoxycholestanol^c^	5,6β-EC	5,6β-Epoxycholesterol; cholesterol β-oxide	5β,6β-epoxy-5β-cholestan-3β-ol	Primary	LMST01010010	108109
7α,25-Dihydroxycholesterol	7α,25-diHC	7α,25-DHC	cholest-5-en-3β,7α,25-triol	Secondary	LMST04030166	11954197
25-Hydroxycholesterol 3-sulfate	25HC3S	25-Hydroxycholesterol sulfate	cholest-5-en-3β,25-diol 3-sulfate	Secondary	LMST05020064	11583880
Cholestane-3β,5α,6β-triol	CT	Cholestanetriol; 5α,6β-dihydroxycholestanol	cholestane-3β,5α,6β-triol	Secondary	LMST01010052	91498
Dendrogenin A	DDA	Dendrogenin A	5α-hydroxy-6β-[2-(1H-imidazol-4-yl)-ethylamino]-cholestan-3β-ol	Secondary	LMST05050025	9806490
7-Keto,27-hydroxycholesterol^b^	7-keto,27-HC	7-Keto-27-HC; 7-keto-26-hydroxycholesterol; 26-hydroxy-7-oxocholesterol	(25R)-3β,26-dihydroxycholest-5-en-7-one	Higher-order	LMST04030180	75964209
7β,27-Dihydroxycholesterol^b^	7β,27-diHC	7β,26-Dihydroxycholesterol	(25R)-cholest-5-en-3β,7β,26-triol	Higher-order	LMST04030178	52931518
Oncosterone^d^	OCDO	Cholestan-6-oxo-3,5-diol; 6-oxo-3,5-diol; 3β,5α-dihydroxycholestan-6-one	6-oxo-cholestan-3β,5α-diol	Higher-order	LMST01010126	24779592
27-Hydroxy-OCDO^b^	27H-OCDO	27-Hydroxy-oncosterone	(25R)-6-oxo-cholestan-3β,5α,26-triol	Higher-order	LMST01010580	171117793

a Metabolic position refers to the pathways discussed in this Review and is not intended as a universal chemical classification. Primary metabolites are generated through a first oxidative transformation of cholesterol or another sterol precursor, whereas secondary and higher-order metabolites arise through subsequent transformations within the pathways considered here. Primary metabolites may arise through enzymatic or nonenzymatic reactions.

b The historically established designation 27-hydroxycholesterol (27-HC) is retained throughout this Review, together with the corresponding 27-hydroxy terminology for its downstream metabolites, to maintain consistency with much of the biological literature. According to systematic sterol numbering, however, the predominant naturally occurring stereoisomer of 27-HC is (25R)-cholest-5-en-3β,26-diol, and the corresponding derivatives are therefore systematically designated as 26-hydroxylated sterols. Accordingly, 7-keto,27-HC and 7β,27-diHC correspond to 7-keto,26-HC/26-hydroxy-7-oxocholesterol and 7β,26-diHC, respectively.

c The historically established terms 5,6α- and 5,6β-epoxycholesterol remain widely used in the literature and databases. Because epoxidation of the Δ5 double bond removes the cholest-5-ene unsaturation, 5,6-epoxycholestanol is used throughout this Review as the structurally appropriate designation. The configurations of the α- and β-cholesterol oxides were assigned by chemical correlation and subsequently established directly by X-ray crystallography.

d Oncosterone (OCDO) is systematically designated 6-oxo-cholestan-3β,5α-diol. The compound has historically also been designated cholestan-6-oxo-3,5-diol, 6-oxo-3,5-diol, and 3β,5α-dihydroxycholestan-6-one.

This definition also requires a strict separation between different levels of evidence. Chemical feasibility does not establish endogenous existence; analytical annotation does not establish structural identity; endogenous occurrence does not establish a precursor–product relationship; enzyme dependence does not quantify metabolic flux; and biological activity observed after exogenous administration does not establish physiological or pathological relevance. Predicted structures, analytical features, structurally authenticated metabolites, metabolically connected products, and biologically relevant mediators therefore represent distinct experimental categories rather than successive labels that can be assigned by inference.

High-resolution mass spectrometry, isotope tracing, spatial metabolomics, chemical synthesis, and computational approaches now provide the means to interrogate this metabolic space at a depth that was previously difficult to achieve. The central challenge is therefore no longer simply to detect additional oxygenated sterols, but to determine what they are, how they are formed, where they are produced, how rapidly they are generated and consumed, and whether they contribute causally to biological processes. In this Review, representative pathways are consequently used not to provide another catalog of established oxysterols, but to define an experimental framework through which the oxysterolome can be discovered, structurally authenticated, metabolically reconstructed, and biologically validated.

## Primary oxysterols as substrates for further metabolism

The first oxygenation of cholesterol does not necessarily define the endpoint of an oxysterol pathway. Primary oxysterols retain functional groups that can undergo further enzymatic transformation, and the resulting products may themselves become substrates for subsequent reactions. Hydroxylation, oxidation, reduction, hydrolysis, and conjugation reactions—including sulfation, glucuronidation, esterification, and amination—can therefore extend an initial oxygenation event into branched, sequential, convergent, or reversible pathways. From this perspective, some primary oxysterols are better considered as metabolic nodes whose fate depends on the enzymatic environment in which they are produced (Fig. 1).

**Fig. 1:**
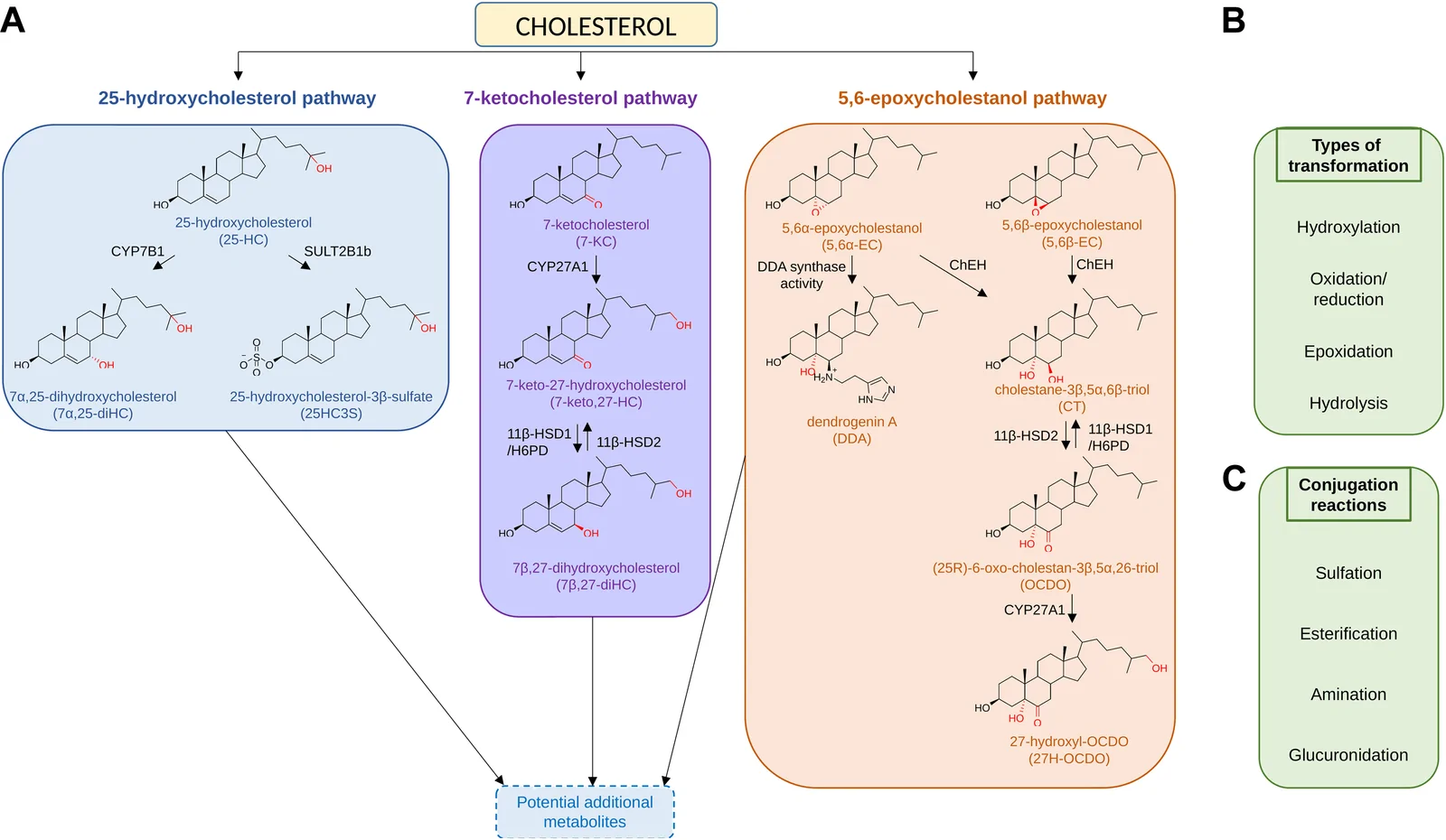
Iterative metabolism generates chemical diversity within the oxysterolome. A: Representative cholesterol-derived pathways illustrate branching, sequential metabolism, convergence, and reversible interconversion of oxygenated sterols. 25-Hydroxycholesterol (25-HC) undergoes CYP7B1-dependent hydroxylation to 7α,25-dihydroxycholesterol (7α,25-diHC) or SULT2B1b-dependent sulfation to 25-hydroxycholesterol-3β-sulfate (25HC3S). The 7-ketocholesterol (7-KC) undergoes CYP27A1-dependent side-chain hydroxylation to 7-keto,27-hydroxycholesterol (7-keto,27-HC), which is reversibly interconverted with 7β,27-dihydroxycholesterol (7β,27-diHC) through 11β-HSD1/H6PD and 11β-HSD2. 5,6α- and 5,6β-epoxycholestanol (5,6α-EC and 5,6β-EC) are hydrolyzed by cholesterol epoxide hydrolase (ChEH) to cholestane-3β,5α,6β-triol (CT), which is reversibly interconverted with oncosterone (OCDO) by 11β-HSD2 and 11β-HSD1/H6PD. OCDO undergoes further CYP27A1-dependent side-chain hydroxylation to 27-hydroxy-OCDO (27H-OCDO), whereas 5,6α-EC undergoes enzyme-dependent conjugation with histamine to generate dendrogenin A (DDA); the enzyme responsible for DDA formation has not yet been identified. B: Representative transformations contributing to oxysterol diversification include hydroxylation, oxidation/reduction, hydrolysis, and epoxidation. C: Conjugation reactions, including sulfation, glucuronidation, esterification, and amination, can further expand oxysterol chemical diversity. The pathways and reaction classes shown are representative rather than exhaustive; analogous iterative transformations may involve oxygenated cholesterol precursors and other sterols, including phytosterols. Only experimentally supported metabolic relationships are shown in panel A.

25-Hydroxycholesterol (25-HC) provides a well-characterized example of metabolic branching. Cholesterol 25-hydroxylase (CH25H) converts cholesterol to 25-HC, although other enzymatic routes can contribute to its formation (12). 25-HC can subsequently undergo CYP7B1-dependent 7α-hydroxylation to generate 7α,25-diHC, or sulfation by SULT2B1b to form 25-HC 3β-sulfate (25HC3S) 13, 14, 15. Importantly, 7α,25-diHC can also be formed through an alternative sequence involving 25-hydroxylation of 7α-hydroxycholesterol (7α-HC), a reaction catalyzed by CYP3A4 in humans (16). Thus, the same bioactive oxysterol can be reached through alternative sequences of hydroxylation. Further metabolism connects this branch to C25-hydroxylated bile-alcohol metabolism. Among the products associated with this pathway, 5β-cholestane-3α,7α,12α,25-tetrol can be converted to cholic acid in humans (17), while its 3α-glucuronide has been identified as a major bile-alcohol glucuronide in cerebrotendinous xanthomatosis (18). Sulfation can also alter the biological response associated with the precursor. In hepatocytes, 25-HC and 25HC3S were reported to exert opposing effects on lipid-metabolic and inflammatory pathways, including LXR/SREBP and IκBα/NF-κB signaling (19). These observations illustrate how a single primary oxysterol can feed chemically distinct branches through modification at different positions of the sterol scaffold, while a receptor-active downstream product can itself enter further metabolic and conjugative pathways.

Sequential metabolism can also recruit oxysterols initially generated by nonenzymatic oxidation. 7-Ketocholesterol (7-KC), a major product of cholesterol oxidation, is a substrate for CYP27A1, which hydroxylates its side chain to generate 7-keto,27-hydroxycholesterol (7-keto,27-HC; systematically, 7-keto,26-HC) 20, 21. 7-Keto,27-HC can then be reduced by 11β-hydroxysteroid dehydrogenase type 1 (11β-HSD1) to 7β,27-dihydroxycholesterol (7β,27-diHC), while 11β-HSD2 catalyzes the reverse oxidation (22). This sequence illustrates how nonenzymatic cholesterol oxidation can provide substrates for subsequent enzymatic metabolism and how a downstream pair of oxysterols can be maintained through reversible interconversion.

The 5,6-epoxycholestanol pathway provides a particularly illustrative example of extensive metabolic branching. Both 5,6α- and 5,6β-epoxycholestanol (5,6α-EC and 5,6β-EC) are hydrolyzed by cholesterol epoxide hydrolase (ChEH) to cholestane-3β,5α,6β-triol (CT) 23, 24. However, epoxide hydrolysis is not the only metabolic fate of 5,6-ECs. Early isotope-tracing experiments demonstrated that orally administered [4–^14^C]cholesterol-α-epoxide can be converted in rats into radiolabeled bile acids, including hyodeoxycholic and lithocholic acids (25). CT itself can also enter bile acid metabolism. Early metabolic studies were subsequently complemented by structural and sterolomic studies establishing CT-derived acidic metabolites and atypical bile acids in humans 26, 27, 28, 29, 30. More recently, CYP27A1 was shown to generate 27-hydroxylated derivatives of 5,6-ECs, CT and OCDO, and these metabolites were identified in humans (31). CT can undergo additional transformations, including oxidation by 11β-HSD2 to OCDO and sulfation to cholestane-5α,6β-diol-3β-sulfate; the latter branch has recently been revisited using a non-hydrolyzable sulfonate analog of cholestane-5α,6β-diol-3β-sulfate (32). OCDO can subsequently undergo CYP27A1-dependent hydroxylation to 27H-OCDO, whereas, in a chemically distinct branch, 5,6α-EC undergoes stereoselective conjugation with histamine to generate dendrogenin A (DDA) (33). Together, these transformations identify 5,6-ECs and CT as branching metabolic nodes connecting epoxide hydrolysis, side-chain oxidation, bile acid metabolism, sulfation, oxo-sterol formation, and amino-conjugation. These examples reveal recurring forms of organization within oxysterol metabolism. A metabolite may branch into several products, as for 25-HC; undergo sequential transformations, as in the pathway initiated by 7-KC; participate in reversible interconversion, as for 7-keto,27-HC and 7β,27-diHC; or arise through convergence of related precursors, as for CT formation from the two 5,6-EC epimers. The terms primary, secondary, and higher-order used here describe the position of metabolites within such pathways and are not intended as a universal chemical classification (Table 1). Their value is to make explicit that oxygenation products conventionally considered individually may belong to larger metabolic networks whose organization cannot be inferred from their concentrations alone.

## Iterative metabolism expands chemical and functional diversity

Successive metabolism necessarily expands the chemical diversity of oxygenated sterols and their metabolic descendants, but its functional consequences are less predictable. Further transformation may generate a new receptor-active metabolite, reversibly control the availability of a bioactive ligand, produce functional divergence within a branched pathway, or facilitate transport and elimination without creating a new signaling function (Fig. 2). Metabolic depth therefore describes pathway position, not biological importance. Functional consequences must be established for individual precursor–product relationships.

**Fig. 2:**
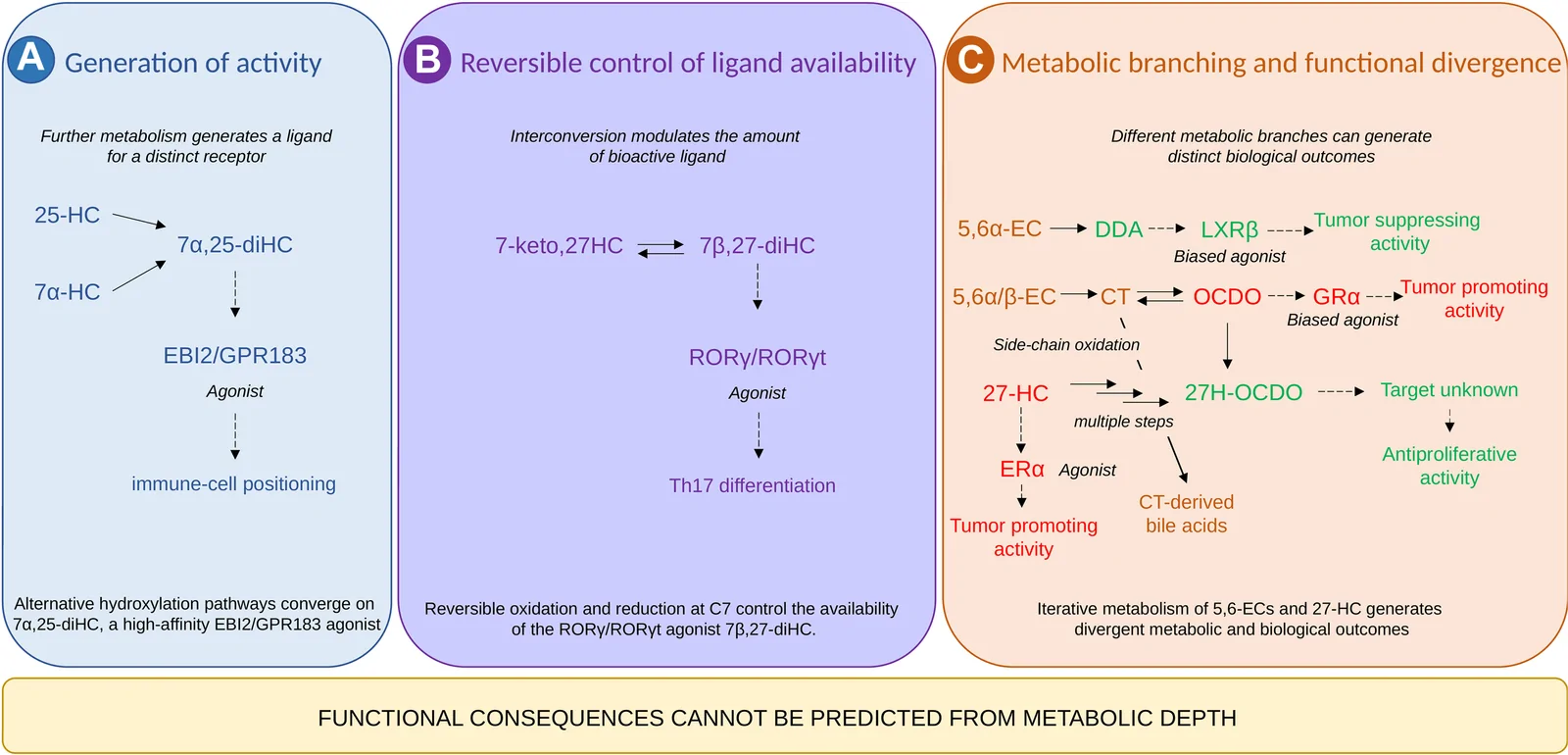
Functional consequences of iterative oxysterol metabolism. Representative pathways illustrate distinct consequences of further oxysterol metabolism. A: Generation of activity. 7α,25-Dihydroxycholesterol (7α,25-diHC) can be generated through alternative hydroxylation sequences, by CYP7B1-dependent 7α-hydroxylation of 25-hydroxycholesterol (25-HC) or by CYP3A4-dependent 25-hydroxylation of 7α-hydroxycholesterol (7α-HC) in humans. These convergent routes generate the same high-affinity agonist of EBI2/GPR183, which regulates immune-cell positioning. B: Reversible control of ligand availability. 7-Keto,27-hydroxycholesterol (7-keto,27-HC) and 7β,27-dihydroxycholesterol (7β,27-diHC) are reversibly interconverted, thereby regulating the availability of 7β,27-diHC, an agonist of RORγ/RORγt that promotes Th17 differentiation. C: Metabolic branching and functional divergence. Iterative metabolism of cholesterol 5,6-epoxides (5,6-ECs) generates metabolites with distinct metabolic and biological outcomes. Hydrolysis of 5,6-ECs generates cholestane-3β,5α,6β-triol (CT), which can undergo further side-chain oxidation and metabolism toward CT-derived bile acids. CT is also reversibly interconverted with the GRα-biased agonist oncosterone (OCDO), which has tumor-promoting activity, while further metabolism of OCDO generates 27H-OCDO, which displays antiproliferative activity through a molecular target that remains unresolved. In a distinct branch, 5,6α-EC is converted to dendrogenin A (DDA), an LXRβ-biased agonist with tumor-suppressive activity. 27H-OCDO can also arise from 27-hydroxycholesterol (27-HC) through a multistep metabolic sequence, whereas 27-HC itself acts as an ERα agonist with tumor-promoting activity. Together, these examples illustrate that iterative oxysterol metabolism can generate receptor activity, produce the same bioactive metabolite through convergent routes, regulate ligand availability, diversify biological function, or redirect metabolites toward further catabolism. Functional consequences therefore cannot be predicted from metabolic depth alone.

The alternative fates of 25-HC provide a clear example. CYP7B1-dependent 7α-hydroxylation of 25-HC generates 7α,25-diHC, a high-affinity endogenous agonist of EBI2/GPR183 that directs B-cell and other immune-cell migration 1, 2; 7α,25-diHC can also be generated through 25-hydroxylation of 7α-hydroxycholesterol (16). Sulfation of 25-HC by SULT2B1b instead generates 25HC3S. Experimental studies have linked 25HC3S to regulation of hepatic lipid metabolism and cell proliferation and to modulation of the LXR-SREBP pathway 34, 35. These effects do not establish 25HC3S as a conventional direct LXR ligand, and its immediate molecular target remains incompletely defined. Nevertheless, the two branches demonstrate that modification of the same primary oxysterol can generate metabolites with markedly different biological properties.

Reversible metabolism provides another mechanism for controlling biological activity. 7β,27-diHC is a direct agonist of RORγ/RORγt and promotes differentiation of Th17 cells (36). Its availability is regulated through interconversion with 7-keto,27-HC by 11β-HSD1 and 11β-HSD2, and manipulation of these enzymes modifies RORγ-dependent responses (22). The relevant biological variable is therefore not simply the abundance of either sterol, but the enzymatically controlled relationship between them. This provides an example in which iterative metabolism regulates access of an endogenous sterol to a receptor-active state.

Further metabolism does not, however, necessarily generate a signaling molecule. The fate of 24(S)-HC provides an important counterpoint. In addition to undergoing further oxidation and conversion toward bile acid products, 24(S)-HC can undergo extensive conjugation. Meng *et al*. identified (24S)-24-hydroxycholesterol 3-sulfate, 24-glucuronide in serum and urine, together with related doubly conjugated oxygenated sterols, demonstrating that sequential sulfation and glucuronidation can substantially alter the chemical properties of an oxysterol (8). In this context, increased polarity is consistent with sterol handling and elimination rather than evidence for acquisition of a new signaling function. This distinction is important when considering the potentially large number of conjugated species that may contribute to the oxysterolome.

Other pathways show that comparatively small structural changes can nevertheless accompany substantial changes in biological activity. Within the 5,6-epoxycholestanol pathway, conversion of CT to OCDO generates a metabolite with GR-dependent tumor-promoting activity, whereas further metabolism generates 27H-OCDO. This metabolite can arise through CYP27A1-dependent hydroxylation of OCDO or through the multistep metabolism of 27-HC and opposes OCDO- and 27-HC-associated proliferative effects in experimental models 31, 37. The molecular target responsible for 27H-OCDO activity remains unresolved, so this relationship should not be interpreted as receptor switching or direct antagonism of OCDO at GR. Separately, conjugation of 5,6α-EC with histamine produces DDA, a biased LXR agonist associated with tumor-cell differentiation and LXRβ-dependent responses 33, 38, 39, 40, 41. The functional dimension of the oxysterolome must therefore be mapped independently from its chemical architecture.

The relationship between metabolic transformation and biological activity is therefore nondirectional. Iterative metabolism can generate activity, alter the balance between receptor-active and less active forms, produce a metabolite with biological effects different from those of its precursor, or contribute predominantly to transport and clearance. Nor does the number of metabolic steps provide a measure of physiological importance: a primary oxysterol may be a potent signaling molecule, whereas a structurally elaborate higher-order product may primarily represent a route of elimination. Chemical and functional diversification should therefore not be assumed to progress in parallel.

This separation between chemical and functional diversity has an important experimental consequence. Theoretical enumeration of possible transformations can estimate the potential dimensions of oxysterol chemical space, but it cannot determine which metabolites exist endogenously or which of them have biological functions. Conversely, the discovery of biological activity for a synthetic or exogenously administered oxysterol does not establish that the corresponding pathway operates at relevant endogenous concentrations. The size of the oxysterolome therefore depends on the level at which it is defined, a question considered in the next section.

## How large is the oxysterolome?

The size of the oxysterolome is unknown and is likely to extend substantially beyond the oxygenated sterols currently characterized. Successive metabolism of oxygenated derivatives of cholesterol, its biosynthetic precursors, and other biologically encountered sterols, including phytosterols, can generate numerous products through hydroxylation, oxidation, reduction, hydrolysis, and conjugation reactions, including sulfation, glucuronidation, esterification, and amination. This broader chemical scope is consistent with previous analytical definitions of oxysterol metabolomes that encompass oxygenated derivatives of cholesterol precursors and plant sterols (42). Because these products may themselves become substrates for further reactions, iterative metabolism can rapidly expand the theoretical chemical space associated with sterol oxygenation. Established pathways already illustrate this principle, including the successive oxidation and conjugation of 24(S)-HC 8, 9, CYP27A1-dependent metabolism of 7-KC 20, 21, the branched metabolism of 25-HC 13, 15, and the successive transformations of 5,6-ECs described previously 23, 31, 33, 37.

The resulting theoretical chemical space is potentially large, particularly when positional and stereochemical isomers and conjugated metabolites are considered. Its dimensions cannot, however, be inferred simply by enumerating chemically plausible transformations. Enzyme specificity, stereochemistry, substrate availability, compartmentalization, competing pathways, and metabolite stability constrain which reactions are biologically realized. The endogenous oxysterolome is therefore expected to represent only a subset of the theoretical chemical space generated by sterol oxygenation and subsequent metabolism.

The challenge is consequently not to assign a definitive number to the oxysterolome, but to determine which members of this potentially large chemical space are actually produced in biological systems. In the framework proposed here, membership in the endogenous oxysterolome requires structural authentication and demonstration of endogenous occurrence. A chemically plausible structure or an analytical feature alone does not satisfy these criteria.

The experimental definition of individual members can therefore be separated from reconstruction of the metabolic network in which they participate. Establishing precursor–product relationships defines metabolic connectivity, whereas identification and perturbation of the enzymes responsible for these transformations establish pathway assignment with increasing levels of confidence. Isotope tracing and kinetic analyses can determine metabolic flux, while tissue, cellular, and subcellular localization define the spatial organization of the network. These levels of information progressively increase the resolution with which the endogenous oxysterolome can be described without changing the fundamental criteria by which a metabolite is assigned to it. The distinct levels of metabolic evidence available for representative pathways are summarized in Supplementary Table S2.

Biological function constitutes a separate dimension of characterization. An authenticated endogenous metabolite may participate predominantly in transport, compartmentalization, turnover, or elimination, may have a signaling function, or may have no biological activity yet identified. Conversely, biological activity demonstrated for a chemically synthesized candidate predicted to arise from sterol metabolism does not establish membership in the oxysterolome unless its endogenous occurrence is subsequently demonstrated. Hypothesis-driven discovery nevertheless provides a valid complementary route. DDA, for example, was synthesized and biologically characterized on the hypothesis that it could represent a natural metabolite before its endogenous occurrence and enzyme-dependent formation from 5,6α-EC and histamine were subsequently demonstrated 33, 43. Thus, synthesis and biological characterization can precede endogenous identification, but they cannot substitute for it.

Defining the oxysterolome therefore involves two related but distinct objectives: establishing which oxygenated sterol-derived molecules exist endogenously, and determining how those molecules are metabolically organized and biologically characterized. The first defines membership in the endogenous oxysterolome; the second reconstructs its connectivity, enzyme dependence, flux, spatial organization, and functional annotation. The analytical and experimental requirements for addressing these questions are considered in the following sections.

## Analytical challenges in defining the endogenous oxysterolome

Experimental expansion of the oxysterolome is limited by analytical coverage as much as by biological abundance. Iterative metabolism generates compounds that differ markedly in polarity, charge, stability, chromatographic behavior, and ionization efficiency, while some oxysterols can also be formed or degraded during sample processing. Consequently, methods optimized for established oxysterols cannot be assumed to recover, distinguish, or quantify their downstream metabolites. The following sections focus on practical challenges that become increasingly important as the oxysterolome expands: analytical accessibility, method-dependent coverage, quantitative reliability, control of analytical artifacts, and structural authentication.

### Analytical accessibility across an expanding physicochemical space

Successive oxygenation and conjugation can profoundly alter the analytical behavior of sterols. Sulfation and glucuronidation increase polarity, esterification increases hydrophobicity, and amino-conjugation can introduce positive charge. No single extraction, chromatographic, ionization, or derivatization procedure can therefore provide uniform coverage of these chemically distinct metabolites.

These differences are evident even among closely related oxysterols. Using radiolabeled standards to follow sample preparation, structure-dependent losses were observed for 5,6α-EC, 5,6β-EC, CT, and OCDO, with marked differences in their responses to purification and trimethylsilylation conditions (44). Analytical performance established for one oxysterol therefore cannot be extrapolated reliably to structurally related or downstream metabolites.

Conjugated oxysterols extend the analytical range still further. Dedicated LC–ESI-MS approaches have resolved positional sulfate isomers and more extensively sulfated derivatives of 27-HC (45), while LC-MS/MS methods have been developed for 24(S)-HC and its sulfate and glucuronide conjugates (46). Esterification shifts the analytical problem toward a more hydrophobic domain, and free and fatty acid-esterified oxysterols can constitute quantitatively different pools 47, 48. Amino-sterols represent yet another physicochemical domain for which chromatographic conditions adapted to charge and polarity can be required 49, 50.

The experimentally accessible oxysterolome is therefore a composite of partially overlapping analytical windows. Expanding coverage will require complementary methods selected according to physicochemical class rather than the assumption that a single oxysterolomic workflow provides comprehensive coverage.

### Method-dependent analytical coverage

Derivatization-based methods illustrate particularly well how analytical chemistry defines oxysterol coverage. Enzyme-assisted oxidation followed by Girard reagent charge tagging, developed extensively by Griffiths *et al*., markedly improves electrospray ionization and provides rich MS^n^ structural information for compatible sterols (51). However, its coverage is determined by the functional groups and reactions required by the workflow and therefore represents a defined analytical window rather than universal oxysterol coverage.

GC-MS approaches provide a complementary analytical window and remain particularly powerful for sterols that can be efficiently converted into suitable volatile derivatives. However, derivatization efficiency and stability are structure-dependent. Trimethylsilylation, for example, can produce markedly different recoveries among closely related 5,6-oxygenated sterols (44). Direct LC-MS approaches, negative-ion methods for sulfated sterols, and dedicated workflows for glucuronides or amino-conjugates similarly interrogate different portions of the chemical space.

These methods should therefore be regarded as complementary rather than competing approaches. High sensitivity and structural resolution within a defined analytical domain are distinct from comprehensive metabolome coverage. Mapping the oxysterolome will require explicit characterization of the compounds each method can detect, distinguish, and quantify, together with integration of data obtained through orthogonal analytical strategies.

### Quantitative reliability and analytical artifacts

The analysis of trace oxysterols in the presence of a large excess of cholesterol presents a distinct analytical challenge: sample processing can alter the composition being measured. Pioneering work by Björkhem *et al*. established the importance of controlling ex vivo cholesterol autoxidation and introduced isotope-based approaches to assess processing-induced oxysterol formation 52, 53. Subsequent methodological studies reinforced the importance of internal standards and controlled sample handling for quantitative oxysterol analysis (48).

This issue remains particularly important for oxysterols that can arise through both biological and nonenzymatic processes. Cholesterol 5,6-epoxides, for example, can be generated during cholesterol oxidation but also participate in endogenous metabolic pathways. Their susceptibility to ex vivo formation does not exclude biological occurrence; rather, it requires experimental determination of the contribution introduced during collection, storage, extraction, and analysis. Preanalytical studies have demonstrated the sensitivity of ROS-derived oxysterol measurements to these variables (54). Controlled sample collection and storage, minimization of exposure to conditions favoring oxidation, and the use of isotope-labeled controls can therefore be important for assessing or limiting ex vivo formation.

Stable-isotope-labeled internal standards remain central to quantitative reliability because they can correct for losses during sample preparation and variability in chromatographic and mass-spectrometric response. Ideally, a chemically matched isotopically labeled standard should accompany an analyte throughout the analytical procedure. This requirement becomes difficult to satisfy as structural diversity increases. A labeled primary oxysterol cannot necessarily correct quantitatively for a secondary metabolite, sulfate, glucuronide, ester, or amino-conjugate with different recovery or ionization behavior.

Quantitative expansion of the oxysterolome will therefore require validation at the level of individual analytes or well-defined chemical classes. Apparent presence can result from artifactual formation, whereas apparent absence can result from instability, poor recovery, incomplete derivatization, ion suppression, chromatographic loss, or insufficient sensitivity. Validation should therefore establish recovery, stability, matrix effects, derivatization efficiency where applicable, and sensitivity for individual analytes or chemically defined classes rather than assuming equivalent performance across the oxysterolome.

### From detection to structural authentication

Positional and stereochemical isomerism is a major obstacle to structural assignment in oxysterol analysis. Identical elemental compositions can correspond to multiple hydroxylation sites or stereoisomers, and this ambiguity increases further with iterative oxygenation and conjugation. Accurate mass therefore constrains the elemental composition but rarely establishes a unique oxysterol structure.

Chromatographic behavior and MS/MS or MS^n^ fragmentation can narrow the range of possible assignments, but positional or stereochemical ambiguity may remain. Comparison with authentic standards analyzed under identical conditions provides substantially stronger evidence. For previously uncharacterized metabolites, chemical synthesis consequently becomes part of the discovery process: synthetic standards allow comparison of retention and fragmentation, discrimination among candidate isomers, development of quantitative assays, and subsequent biological testing. When sufficient material is available, NMR and other orthogonal structural approaches can provide additional evidence that cannot be obtained by MS alone.

The distinction between an analytical feature, an annotated candidate, and a structurally authenticated metabolite should therefore be explicit. This principle is consistent with metabolomics identification standards that classify assignments according to the structural evidence available (55). Standardized nomenclature and resources such as LIPID MAPS are equally important because they allow the experimentally achieved level of structural resolution to be reported unambiguously (56). Representative examples of systematic nomenclature and database identifiers are provided in Table 1, while Supplementary Table S1 summarizes their chemical and stereochemical characterization, early identification in biological systems, and metabolic assignment.

Structural discovery may also proceed in the opposite direction. Chemically conceived or predicted metabolites can first be synthesized and analytically characterized, generating reference HRMS, MS/MS, and chromatographic properties that enable their targeted search and subsequent authentication in biological matrices. This strategy was instrumental in the discovery of DDA, which was first synthesized and biologically characterized before being subsequently identified as an endogenous metabolite and assigned to a metabolic pathway 33, 43. Thus, chemical synthesis can contribute not only to structural authentication of an analytically detected candidate but also to hypothesis-driven discovery of previously unrecognized endogenous metabolites. In either discovery route, structural authentication in a biological matrix should be accompanied by controls that distinguish endogenous occurrence from formation during collection, storage, extraction, or analysis, particularly for metabolites susceptible to nonenzymatic oxidation.

## From structural authentication to metabolic causality

Structural authentication establishes what a molecule is but not how it was formed. Two structurally related metabolites detected in the same sample are not necessarily connected through a precursor–product relationship, and conversion of a sterol by an enzyme in vitro does not establish that the reaction contributes substantially to its formation in vivo. Reconstructing the oxysterolome therefore requires experimental evidence for the biochemical connections between authenticated metabolites and, ultimately, for the direction, magnitude, and spatial organization of metabolic flux (Fig. 3).

**Fig. 3:**
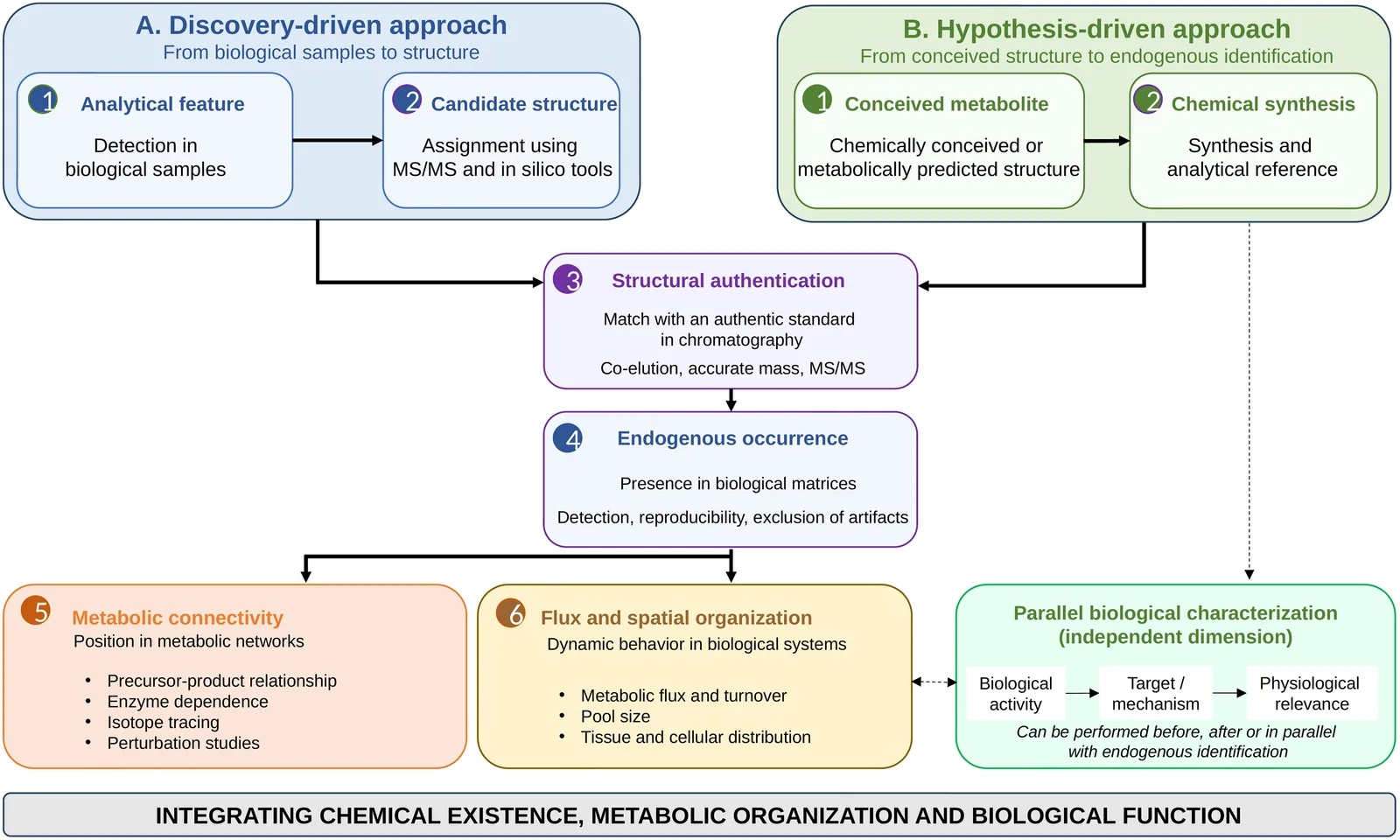
Complementary experimental strategies for defining and reconstructing the endogenous oxysterolome. Members of the oxysterolome can be identified through complementary discovery-driven and hypothesis-driven approaches. A: Discovery-driven approach. Analytical features detected in biological samples can be assigned candidate structures using high-resolution mass spectrometry (HRMS), MS/MS fragmentation, and computational tools. B: Hypothesis-driven approach. Chemically or metabolically plausible structures can be conceived and synthesized before their endogenous occurrence is known. Chemical synthesis provides authentic standards whose analytical properties, including accurate mass, MS/MS fragmentation, and chromatographic behavior, enable targeted searches in biological matrices. Both approaches converge on structural authentication through comparison with authentic standards under matched analytical conditions, including coelution, accurate mass, and MS/MS, complemented when necessary by NMR or other orthogonal structural methods. Endogenous occurrence must then be established with appropriate controls for reproducibility and artifactual formation. Authenticated endogenous metabolites can subsequently be positioned within metabolic networks by establishing precursor–product relationships, enzyme dependence, isotope tracing, and genetic or pharmacological pathway perturbation. Quantitative and kinetic analyses, including isotope tracing, together with spatially resolved approaches, can further define pool size, metabolic flux, turnover, and cellular or tissue organization. Biological characterization represents an independent dimension. In discovery-driven studies, it will generally follow structural authentication and demonstration of endogenous occurrence, whereas in hypothesis-driven studies it may precede endogenous detection. Biological activity alone, however, does not establish membership in the endogenous oxysterolome. Together, these complementary approaches integrate chemical existence, metabolic organization, and biological function without assuming that evidence at one level establishes evidence at another.

### Establishing metabolic connectivity

The most direct evidence for a precursor–product relationship is obtained by tracing an isotopically labeled precursor into a structurally authenticated downstream metabolite. Time-resolved isotope tracing can additionally establish pathway direction and provide information on turnover. Such approaches become increasingly important as iterative metabolism proceeds because structurally identical products may be reached through different reaction sequences.

The metabolism of 24(S)-HC provides a particularly strong example. Its predominant cerebral origin and net export from brain into the circulation were established by arterio-venous measurements in humans 6, 7. Its subsequent metabolic fate was examined using complementary experiments with labeled 24(S)-HC in human hepatocytes, administration of labeled 24(S)-HC to a human volunteer, and characterization of metabolites recovered from ileocecal fluid. These studies demonstrated conversion into bile acids and more highly oxygenated sterols, directly establishing that circulating 24(S)-HC enters successive downstream pathways (9).

Isotope tracing is not the only route to metabolic connectivity. Convergent biochemical and genetic evidence can connect endogenous metabolites when isotope experiments are unavailable. The pathway generating 7α,25-diHC illustrates this approach: genetic deficiency of CH25H or CYP7B1 reduces EBI2 ligand production, whereas loss of HSD3B7 increases ligand abundance by impairing its inactivation, providing complementary genetic evidence for the organization of this metabolic pathway (57). Similarly, conversion of 7-KC into side-chain-oxygenated metabolites has been demonstrated both biochemically and in cellular systems (20). Evidence for connectivity can therefore arise from different experimental designs. Identification of the enzymes responsible for individual transformations provides an additional level of pathway assignment, considered below.

### Enzyme dependence and pathway assignment

Enzyme identification provides an important level of pathway evidence, but different experiments establish different claims. Conversion by a purified or recombinant enzyme demonstrates biochemical competence. Alteration of product abundance following genetic or pharmacological perturbation supports enzyme dependence in a biological system. Demonstration of isotope transfer, loss and rescue of metabolite production, or pathway perturbation in vivo provides stronger evidence that the reaction contributes to endogenous metabolic flux.

The reversible interconversion of 7-keto,27-HC and 7β,27-diHC illustrates this distinction. Human 11β-HSD1 reduces 7-keto,27-HC to 7β,27-diHC, whereas 11β-HSD2 catalyzes the reverse oxidation (22). The two enzymes therefore control the balance between interconnected metabolites rather than simply catalyzing consecutive steps in a unidirectional pathway. This type of reversible relationship is important when reconstructing a metabolic network because pathway direction can depend on enzyme expression, cofactor availability, and cellular context.

Incomplete enzyme assignment should likewise remain explicit. DDA formation provides an example in which the enzymatic nature of a metabolic transformation was established before identification of the enzyme itself. Incubation of [^14^C]-5,6α-EC with histamine and mouse brain homogenates resulted in DDA formation with saturable kinetics, whereas no reaction occurred in the absence of tissue homogenate or after boiling or pronase treatment of the homogenate. The reaction was also stereoselective, as 5,6β-EC did not generate DDA under the same conditions. These experiments established that DDA formation from 5,6α-EC and histamine is enzyme-dependent, although the molecular identity of the enzyme responsible for this conjugation remained unresolved (33). This example illustrates an important distinction in pathway reconstruction: enzymatic dependence can be experimentally established even when the enzyme responsible has not yet been molecularly identified. This pathway can therefore be considered metabolically connected without being enzymatically complete. More generally, pathway reconstruction should distinguish experimentally demonstrated metabolic connectivity from complete molecular identification of the enzymatic machinery responsible for it.

### Pool size is not metabolic flux

Steady-state concentration reflects the balance among formation, consumption, transport, sequestration, and elimination. A rapidly produced metabolite can remain at low abundance when it is efficiently consumed, whereas accumulation may result from impaired downstream metabolism rather than increased synthesis. Concentration alone therefore cannot establish pathway activity.

This distinction is evident in genetic perturbation of oxysterol metabolism. In Cyp7b1-deficient mice, 25-HC and 27-HC accumulate because an important route of their downstream metabolism has been disrupted (58). Their increased pool sizes consequently do not indicate increased production. Conversely, a metabolite undergoing rapid formation and consumption may show little change in steady-state abundance despite substantial flux.

Flux analysis therefore requires information beyond concentration. Stable-isotope tracing, kinetic measurements, precursor enrichment, product appearance, and controlled pathway perturbation can distinguish changes in production from changes in consumption or clearance. This distinction becomes increasingly important in a branched network, where perturbation of one reaction can redistribute material among several downstream products without necessarily producing large changes in the abundance of the upstream metabolite.

### Metabolic networks are spatially organized

A biochemically feasible reaction can operate in vivo only when substrate, enzyme, cofactors, and transport processes are brought into an appropriate spatial context. Tissue distribution, cell-type specificity, subcellular localization, and exchange between organs therefore determine which portions of the oxysterolome are accessible in a given biological system.

24(S)-HC illustrates this organization at the interorgan level: production in the brain, export into the circulation, and subsequent hepatic metabolism connect cholesterol turnover in the central nervous system to sterol metabolism in the liver 6, 7. At a finer spatial scale, 7α,25-diHC metabolism is organized among lymphoid stromal-cell populations through differential expression of enzymes involved in its formation and degradation, thereby generating local control of EBI2 ligand availability (57).

Spatial information can also be obtained directly from regional concentration gradients. Comparison of jugular and peripheral venous plasma in humans revealed significant cerebral efflux of OCDO, estimated at approximately 0.1 mg/day, providing evidence consistent with production within the brain and subsequent export into the circulation (59). Importantly, such a gradient establishes a net regional contribution but does not by itself identify the producing cell type or the complete pathway responsible for metabolite formation.

The oxysterolome should therefore ultimately be represented not simply as a network of chemical transformations but as a spatially and kinetically organized network. Establishing that two metabolites are connected identifies an edge in that network; determining where the reaction occurs and how much material passes through it defines its biological organization. These dimensions are necessary before changes in metabolite abundance can be interpreted mechanistically.

Emerging spatial approaches may progressively extend this analysis from organ-level gradients to tissue and cellular organization. Mass spectrometry imaging is beginning to provide spatially resolved access to sterol metabolism, while continued gains in sensitivity and sample miniaturization may ultimately permit oxysterol analysis at the level of defined cell populations or individual cells (60). At present, however, the low abundance and structural isomerism of many oxysterols remain major limitations to comprehensive spatial and single-cell analysis.

## From endogenous metabolites to biologically relevant mediators

Structural authentication and demonstration of endogenous occurrence establish that a compound is a member of the endogenous oxysterolome, while metabolic connectivity places that metabolite within an experimentally defined pathway or network. Neither establishes that the metabolite contributes to a biological process. Functional relevance requires additional evidence linking the concentrations and sites at which a metabolite is produced to a measurable phenotype and, where possible, to a defined molecular mechanism. These levels of evidence should remain distinct. Endogenous occurrence supports biological plausibility; perturbation of metabolite formation can establish causality; and identification of a molecular target can explain how the metabolite produces its effects. The evidence supporting these different levels of metabolic and functional assignment in representative oxysterol pathways is summarized in Supplementary Table S2.

For discovery-driven expansion of the oxysterolome, structural authentication and demonstration of endogenous occurrence should generally precede extensive functional characterization. A complementary hypothesis-driven route remains possible when a metabolically plausible compound is synthesized and characterized first, but biological activity of such a candidate cannot substitute for subsequent demonstration of endogenous occurrence. These complementary routes are summarized in Figure 3.

### Endogenous occurrence and biological plausibility

Detection of an authenticated metabolite in cells, tissues, or biological fluids is a prerequisite for assigning an endogenous function, but concentration alone is insufficient. Biological plausibility depends on whether the metabolite reaches its putative target at the relevant site, whether endogenous exposure is compatible with concentrations required for activity, and whether its production changes under conditions in which the proposed function is observed.

This issue is particularly important for oxysterols because concentrations measured in whole tissues or plasma may not reflect exposure within a specific cellular or subcellular compartment. Local synthesis, intracellular sequestration, protein or membrane binding, esterification, transport, and rapid metabolism can produce substantial differences between bulk concentration and target exposure. Conversely, biological effects observed after addition of micromolar concentrations of an oxysterol cannot be assumed to occur physiologically when endogenous concentrations are substantially lower, unless local accumulation or another mechanism supporting comparable target exposure is demonstrated.

7α,25-diHC provides a useful example in which endogenous occurrence, enzymatic regulation, and biological potency converge. The metabolite activates EBI2/GPR183 at low concentrations, and genetic manipulation of enzymes controlling its formation and degradation alters EBI2-dependent immune-cell positioning 1, 2, 57. In this case, biological plausibility does not rest solely on detection of an endogenous ligand or on receptor activation by an added compound, but on correspondence between metabolite production, receptor sensitivity, and the phenotype controlled by the pathway.

Endogenous occurrence should therefore be regarded as a necessary but intermediate level of functional evidence. It establishes that a candidate molecule is available to participate in biology; demonstrating that the endogenous pathway actually contributes to a phenotype requires perturbation of its formation or removal.

### From metabolic perturbation to biological causality

A stronger test of biological relevance is obtained when selective manipulation of a metabolic pathway changes both the endogenous metabolite and the associated phenotype. Genetic deletion or silencing of a biosynthetic enzyme, inhibition of metabolite formation, enhancement of degradation, or restoration of the pathway can provide such evidence. The interpretation is strongest when changes in metabolite abundance are measured directly, because enzyme perturbation alone may affect several substrates or products.

The CT-OCDO pathway provides an unusually complete example of this approach. OCDO was identified as an endogenous metabolite of CT, with 11β-HSD2 catalyzing CT oxidation to OCDO and 11β-HSD1 catalyzing the reverse reaction. OCDO concentrations were increased in human breast tumors relative to nontumoral tissue, together with increased expression of enzymes favoring its production. In experimental breast cancer models, pharmacological inhibition of the upstream cholesterol epoxide hydrolase pathway or genetic suppression of 11β-HSD2 decreased OCDO production and inhibited tumor growth (37).

The same study connected metabolic perturbation to the action of the metabolite itself. OCDO stimulated breast cancer cell proliferation and tumor growth through the glucocorticoid receptor (GR/*NR3C1*), while interference with GR signaling suppressed its proliferative effects. Thus, the evidence does not rely on the activity of exogenously administered OCDO alone: human tumor measurements, precursor–product assignment, enzyme identification, metabolic perturbation, receptor dependence, and in vivo tumor phenotypes converge on the same pathway.

This level of convergence provides a useful benchmark for functional assignment within the oxysterolome. Few metabolites will initially satisfy every criterion, and absence of one type of evidence should not automatically exclude biological relevance. However, the distinction between activity of a compound and causality of an endogenous metabolic pathway should remain explicit. Demonstrating that an oxysterol produces a phenotype when added to cells or animals is fundamentally different from demonstrating that changing its endogenous formation modifies that phenotype.

### From biological activity to molecular mechanism

Identification of a molecular target provides a further level of evidence by connecting an endogenous metabolite to a defined signaling mechanism. For several oxysterols, this relationship is well established. 7α,25-diHC is an endogenous agonist of EBI2/GPR183; 7β,27-diHC activates RORγ/RORγt (*RORC*); and 27-HC can act through estrogen receptor alpha (*ESR1*) and LXRs (LXRα/*NR1H3*, LXRβ/*NR1H2*) 2, 36, 61, 62. Such assignments are strongest when direct ligand–target interaction, target-dependent transcriptional or signaling responses, and loss of biological activity after target perturbation are demonstrated together.

Target assignment should nevertheless be distinguished from pathway assignment. A metabolite may have reproducible biological activity without a known direct target, while identification of a receptor activated at high concentrations does not establish that this interaction mediates the endogenous effect. The molecular mechanism should therefore be evaluated against endogenous exposure, binding or activation potency, target expression, and genetic or pharmacological dependence.

DDA illustrates a situation in which metabolic and receptor-level evidence can be combined but should still be described precisely. DDA is generated by enzyme-dependent conjugation of 5,6α-EC with histamine and binds both LXR isoforms, with preferential affinity for LXRβ. Its transcriptional profile differs from that produced by conventional LXR agonists, and LXRβ-dependent responses contribute to tumor-cell differentiation and lethal autophagy 33, 38, 39, 40, 41. DDA is therefore more appropriately described as a biased LXR agonist than as an LXRβ-specific ligand (63).

Conversely, biological activity can be established before the responsible molecular target is known. CYP27A1-dependent hydroxylation of OCDO generates 27H-OCDO, which displays antiproliferative and antitumor properties and can oppose effects associated with OCDO in experimental models, but the molecular target responsible for these effects remains unresolved (31). Assigning receptor switching or direct antagonism of OCDO at GR would therefore go beyond the available evidence.

### Functional consequences of iterative metabolism

The previous examples show that further metabolism has no single functional consequence (Fig. 2). Transformation of a primary oxysterol may generate a receptor ligand, regulate the availability of a receptor-active metabolite through reversible interconversion, produce a compound acting through a different mechanism, or generate more polar products associated predominantly with transport and elimination. Iterative metabolism can therefore generate, preserve, redirect, or terminate biological activity, but none of these outcomes follows automatically from the number of metabolic steps.

Functional annotation of the oxysterolome should therefore proceed independently from reconstruction of its chemical architecture. A metabolite can be structurally authenticated and metabolically connected without having a demonstrated signaling role; conversely, biological activity of a synthesized candidate for a predicted metabolite does not establish that the molecule occurs endogenously. Discovery-driven and hypothesis-driven approaches therefore converge only when chemical identity, endogenous occurrence, and biological evidence are experimentally connected.

This framework also avoids treating biological function as a mandatory endpoint for every member of the oxysterolome. Many metabolites may primarily mediate transport, compartmentalization, turnover, or elimination, while others may acquire signaling functions in particular tissues or physiological states. The objective is therefore not to classify every newly detected oxysterol as a signaling molecule, but to determine experimentally which metabolic transformations alter biological information and under what conditions.

## Conclusions and perspectives

The oxysterolome provides a framework for considering oxygenated sterols as components of interconnected metabolic networks rather than as isolated metabolites. It builds on decades of work establishing the chemical diversity, metabolism, and biological activities of oxysterols, while extending this foundation to the systematic reconstruction of the products generated through successive metabolic transformations. Its scope is therefore not defined by a fixed list of compounds, but by experimentally demonstrated relationships among sterol-derived metabolites generated through successive transformations.

The potential dimensions of this metabolic space are substantially greater than those represented by the oxysterols routinely measured today. Iterative hydroxylation, oxidation, reduction, hydrolysis, and conjugation reactions, including sulfation, glucuronidation, esterification, and amination, can generate numerous candidate structures, but chemical possibility should not be confused with biological existence. The central challenge is to determine which of these candidates occur endogenously, how they are connected, where and at what rates they are produced, and which contribute to biological processes. Meeting this challenge will require complementary analytical methods, authentic standards, isotope tracing, pathway perturbation, and spatially resolved approaches rather than reliance on any single oxysterolomic platform.

An expanded view of oxysterol metabolism may also change the interpretation of metabolite measurements. The concentration of an individual oxysterol reflects its position within a network of formation and consumption and cannot, by itself, define pathway activity. Changes associated with physiology or disease may therefore result from altered metabolic flux, redistribution among competing branches, impaired downstream metabolism, or changes in transport and compartmentalization. Reconstructing these relationships should provide a more mechanistic interpretation of oxysterol profiles than measurements of individual metabolites considered in isolation.

The same caution applies to biological function. The consequences of further metabolism are context-dependent and cannot be inferred from pathway position or structural complexity. Some downstream metabolites may act as signaling molecules, whereas others may primarily contribute to sterol transport, turnover, or elimination. Functional annotation must therefore remain experimentally separable from chemical and metabolic annotation.

Defining the oxysterolome will ultimately require a transition from cataloging oxygenated sterols to reconstructing experimentally validated metabolic networks in which molecular identity, pathway connectivity, flux, and biological causality are resolved together.

## Declaration of generative AI and AI-assisted technologies in the writing process

During the preparation of this work, the authors used ChatGPT (OpenAI) to improve the clarity and readability of the article. After using this tool, the authors reviewed and edited the content as needed and take full responsibility for the content of the published article.

## Supplemental data

This article contains supplemental data.

## Conflict of interest

The authors declare that they have no conflicts of interest with the contents of this article.

## Supplemental data

Supplementary Table S2

Supplementary References
