# Supplementary material for: Defining the oxysterolome: analytical coverage, metabolic connectivity, and biological validation
Source: J Lipid Res. 2026 Oct 12;67(11):101170. doi: 10.1016/j.jlr.2026.101170 (PMC13761991; doi:10.1016/j.jlr.2026.101170)
Supplement: Supplementary file 1 [file mmc1.docx]

**Supplementary Table S1.** **Chemical identity, historical characterization, and metabolic assignment of representative metabolites within the oxysterolome.**

| Metabolite | Formula / exact mass (Da)* | LIPID MAPS ID / PubChem CID | Chemical identification or preparation | Structural / stereochemical authentication | Early identification in a biological system | Metabolic origin / pathway assignment |
| --- | --- | --- | --- | --- | --- | --- |
| 7α-HC | C27H46O2 / 402.3498 | LMST01010013 / 107722 | 7α-HC obtained by reduction of the 7α-hydroperoxide generated during cholesterol oxidation [1] | Identity established by classical sterol chemistry [1] | Reported in animal biological material in 1953 [2] | Cholesterol → 7α-HC through CYP7A1 [3] |
| 24(S)-HC | C27H46O2 / 402.3498 | LMST01010019 / 121948 | 24-HC epimers prepared and compared with cerebrosterol [4] | Early C24 configurational assignment based on molecular rotation [5]; absolute configuration of natural cerebrosterol established as 24S by chemical correlation [6] | Cerebrosterol isolated from horse brain [4,7] and subsequently from human brain [8] | Cholesterol → 24(S)-HC through CYP46A1; cerebral export into circulation [9,10] |
| 25-HC | C27H46O2 / 402.3498 | LMST01010018 / 65094 | Independently synthesized as cholest-5-en-3β,25-diol in 1950 [11,12] | Structure established by synthesis and derivative characterization [11,12] | Identified as a cholesterol-derived metabolite in mammalian systems [13] | Cholesterol → 25-HC through CH25H [14]; additional formation routes contribute |
| 27-HC† | C27H46O2 / 402.3498 | LMST01010088 / 123976 | 5-Cholestene-3β,26-diol chemically synthesized [15] | Side-chain structure established; predominant natural stereoisomer corresponds to (25R)-26-HC [15,16] | Identified as a mammalian cholesterol metabolite [13] | Cholesterol → 27-HC through CYP27A1; human CYP27A1 molecularly characterized [17] |
| 7-KC | C27H44O2 / 400.3341 | LMST01010049 / 91474 | Free 7-KC described by Mauthner and Suida and subsequently characterized as a major cholesterol autoxidation product [18,19] | Established by classical chemistry and comparison with authentic material [19] | Early reports of cholesta-3,5-dien-7-one in human atherosclerotic aortas provided indirect historical evidence [20,21]; 7-KC was subsequently directly identified in human atheromatous plaques using chromatography/MS and authentic material [22] | Formed through non-enzymatic and enzymatic processes; further metabolized by CYP27A1 [23,24] |
| 5,6α-EC‡ | C27H46O2 / 402.3498 | LMST01010011 / 227037 | α-Cholesterol oxide prepared by peracid oxidation [25] | Relative stereochemistry assigned chemically [26]; three-dimensional structure established by X-ray crystallography [27] | Identified in human serum [28] | Enzymatic and non-enzymatic formation; hydrolyzed to CT by ChEH [29,30,31] |
| 5,6β-EC‡ | C27H46O2 / 402.3498 | LMST01010010 / 108109 | Pure β-cholesterol oxide prepared chemically [32,33] | Relative stereochemistry assigned chemically [26]; three-dimensional structure established by X-ray crystallography [27] | Formation demonstrated in rat liver subcellular fractions [30] | Oxidative formation; hydrolyzed to CT by ChEH [30,31] |
| 7α,25-diHC | C27H46O3 / 418.3447 | LMST04030166 / 11954197 | Prepared in synthetic studies [34,35] | Structural and stereochemical identity established synthetically [34,35] | Formation from 25-HC demonstrated in rat, pig and human liver microsomes [36] | 25-HC → 7α,25-diHC through oxysterol 7α-hydroxylation, subsequently assigned to CYP7B1 [36,37] |
| 25HC3S | C27H46O5S / 482.3066 | LMST05020064 / 11583880 | Identified as a sulfated derivative of 25-HC [38] | Characterized by MS and desulfation approaches [38,39] | Identified in hepatocyte-derived material [38] | 25-HC → 25HC3S through PAPS-dependent SULT2B1b activity [39] |
| CT | C27H48O3 / 420.3603 | LMST01010052 / 91498 | Obtained by oxidation of cholesterol with hydrogen peroxide [40] | Stereochemistry established by classical chemical studies [26,41] | Identified by MS as a metabolite after administration of epoxycholesterol to rats [42] | Both 5,6-EC epimers → CT through ChEH [30]; EBP is the catalytic component and DHCR7 a regulatory component [31] |
| DDA | C32H55N3O2 / 513.4294 | LMST05050025 / 9806490 | Initially prepared as a synthetic histamine–sterol conjugate [43] | Identity of endogenous DDA established by comparison with synthetic material [43,44] | Identified as an endogenous mammalian metabolite [44] | Enzyme-dependent conjugation of 5,6α-EC with histamine; catalytic enzyme remains unresolved [44] |
| 7-keto,27-HC† | C27H44O3 / 416.3290 | LMST04030180 / 75964209 | Generated and characterized in metabolic studies [23,24] | Identity established by comparison with authentic material and modern MS approaches [24,45] | Identified in human atherosclerotic material and related biological samples [23,45] | 7-KC → 7-keto,27-HC through CYP27A1 [23,24]; interconverted with 7β,27-diHC through 11β-HSDs [46] |
| 7β,27-diHC† | C27H46O3 / 418.3447 | LMST04030178 / 52931518 | 7β-Hydroxylated sterol characterized by synthetic and analytical approaches [46,47] | Structural assignment supported by synthetic and modern analytical approaches [46,47] | 7β-Hydroxylated products generated from 27-HC reported in human liver preparations [48] | Interconversion with 7-keto,27-HC controlled by 11β-HSD1/11β-HSD2 [46] |
| OCDO§ | C27H46O3 / 418.3447 | LMST01010126 / 24779592 | Prepared from cholesterol by N-bromosuccinimide oxidation [49] | Chemical identity supported by derivative characterization and comparison with previously described material [49] | Identified as a major neutral metabolite of CT in rats [50]; evidence consistent with cerebral production and export in humans [51] | CT → OCDO through 11β-HSD2; OCDO → CT through 11β-HSD1 [52] |
| 27H-OCDO† | C27H46O4 / 434.3396 | LMST01010580 / 171117793 | Identified as a hydroxylated derivative of OCDO [53] | Characterized by comparison with authentic material, chromatography and MS [53] | Identified as an endogenous mammalian metabolite [53] | OCDO → 27H-OCDO through CYP27A1 [53] |

Footnotes to Table S1

* Exact masses correspond to neutral molecules.

† The historically established designation 27-hydroxycholesterol (27-HC) is retained throughout this Review, together with the corresponding 27-hydroxy terminology for its downstream metabolites, to maintain consistency with much of the biological literature. According to systematic sterol numbering, however, the predominant naturally occurring stereoisomer of 27-HC is (25R)-cholest-5-en-3β,26-diol, and the corresponding derivatives are therefore systematically designated as 26-hydroxylated sterols. Accordingly, 7-keto,27-HC and 7β,27-diHC correspond to 7-keto,26-HC/26-hydroxy-7-oxocholesterol and 7β,26-diHC, respectively.

‡ The historically established terms 5,6α- and 5,6β-epoxycholesterol remain widely used in the literature and databases. Because epoxidation of the Δ5 double bond removes the cholest-5-ene unsaturation, 5,6-epoxycholestanol is used throughout this Review as the structurally appropriate designation. The configurations of the α- and β-cholesterol oxides were assigned by chemical correlation by Plattner and Lang in 1944; their three-dimensional structures were subsequently established directly by X-ray crystallography by Paillasse et al. in 2012 [27].

§ Oncosterone (OCDO) is systematically designated 6-oxo-cholestan-3β,5α-diol. The compound has historically also been designated cholestan-6-oxo-3,5-diol, 6-oxo-3,5-diol, and 3β,5α-dihydroxycholestan-6-one.
