# Supplementary material for: Defining the oxysterolome: analytical coverage, metabolic connectivity, and biological validation
Source: J Lipid Res. 2026 Oct 12;67(11):101170. doi: 10.1016/j.jlr.2026.101170 (PMC13761991; doi:10.1016/j.jlr.2026.101170)
Supplement: Supplementary file 2 [file mmc2.docx]

**Supplementary Table S2. Experimental evidence supporting metabolic and functional assignment of representative pathways within the oxysterolome**

| Metabolic relationship | Endogenous occurrence of product | Structural identity | Precursor–product relationship | Enzyme/pathway assignment | Flux/tracing/spatial evidence | Biological activity | Molecular target/mechanism | Evidence remaining to be resolved | Key primary reference(s) |
| --- | --- | --- | --- | --- | --- | --- | --- | --- | --- |
| Cholesterol → 7α-HC | Established | Established | Established | Established: CYP7A1 | Supported | Established | Direct RORα/RORγ ligand with ROR-dependent transcriptional effects; also a major intermediate in classical bile-acid synthesis | Physiological contribution of direct ROR signaling relative to its role as a rapidly metabolized bile-acid precursor | [2,3,54] |
| Cholesterol → 24(S)-HC | Established | Established | Established | Established: CYP46A1 | Established: cerebral production and brain-to-circulation export demonstrated in humans | Established | Regulation of sterol homeostasis; LXR-dependent activities documented | Relative contribution of receptor-dependent activities across neural cell types and physiological or pathological contexts | [9,10,55] |
| Cholesterol → 25-HC | Established | Established | Established | Established: CH25H; additional routes contribute | Supported/context-dependent | Established | Regulation of SREBP processing through SCAP/INSIG; LXR-dependent and additional biological activities | Quantitative contribution of individual formation routes across tissues and conditions | [13,14,56] |
| Cholesterol → 27-HC† | Established | Established | Established | Established: CYP27A1 | Established | Established | ER-dependent/SERM and LXR-dependent activities | Tissue-specific balance among formation, further metabolism, export, and receptor-dependent activities | [13,17,57,58] |
| 25-HC → 7α,25-diHC | Established | Established | Established | Established: CYP7B1 | Supported | Established | EBI2/GPR183 agonist; controls immune-cell migration | Quantitative pathway flux and its regulation across tissues and immune contexts | [36,37,59,60] |
| 25-HC → 25HC3S | Established | Established | Established | Established: SULT2B1b; PAPS-dependent sulfation | Supported | Established in experimental systems: regulation of lipid metabolism and hepatocyte proliferation | Supported: modulation of LXR–SREBP signaling; direct molecular target remains incompletely defined | Direct molecular target(s); quantitative endogenous flux; relative contributions of 25-HC depletion and 25HC3S formation | [38,39,61,62] |
| Cholesterol → 5,6α-EC / 5,6β-EC | Established | Established | Established | Partial/context-dependent: enzymatic and non-enzymatic formation | Partial/context-dependent | Established in experimental systems | Multiple biological activities have been reported; no single molecular mechanism accounts for both epimers across biological systems | Relative contributions of enzymatic and non-enzymatic formation in vivo and epimer-specific flux | [28,29,30] |
| 5,6α-EC / 5,6β-EC → CT | Established | Established | Established | Established: ChEH; EBP catalytic component and DHCR7 regulatory component | Supported | Established in experimental systems; attribution may be influenced by further metabolism | No single direct molecular target established; CT is also a metabolic precursor of OCDO | Direct CT-dependent mechanisms versus effects mediated by downstream metabolism; quantitative CT→OCDO flux across tissues | [30,31,42,63] |
| 5,6α-EC + histamine → DDA | Established | Established | Established | Partial: enzyme-dependent conjugation established; catalytic enzyme identity unresolved | Supported | Established: tumor-cell differentiation, tumor-suppressive activity, lethal autophagy, and generation of immunogenic sEV | Established: biased LXR agonist, with LXRβ-dependent differentiation, autophagic, and immunomodulatory responses | Identity of the DDA-synthesizing enzyme; quantitative endogenous flux and tissue-specific regulation | [44,64,65] |
| 7-KC → 7-keto,27-HC† | Established | Established | Established | Established: CYP27A1 | Supported | Partial/context-dependent | Biological effects reported, but functional attribution to 7-keto,27-HC itself is complicated by its further metabolism | Physiological function of 7-keto,27-HC itself versus its role as a metabolic intermediate; quantitative conversion to downstream products | [23,24,45] |
| 7-keto,27-HC ⇄ 7β,27-diHC† | Established | Established | Established | Established: 11β-HSD1 catalyzes reduction to 7β,27-diHC; 11β-HSD2 catalyzes the reverse oxidation | Supported | Established for 7β,27-diHC | Established for 7β,27-diHC: RORγ/RORγt agonist promoting Th17 differentiation | Quantitative in vivo contribution of reversible 11β-HSD-dependent interconversion and tissue-specific regulation | [46,48,66] |
| CT ⇄ OCDO | Established: OCDO detected endogenously, increased in human breast tumors; evidence also supports cerebral production/export in humans | Established | Established | Established: 11β-HSD2 catalyzes CT→OCDO; 11β-HSD1 catalyzes OCDO→CT | Supported: experimental metabolic conversion; human arterio-venous evidence supports a cerebral contribution to circulating OCDO | Established: tumor-promoting activity | Established: GR/NR3C1-dependent proliferative signaling | Quantitative contribution and regulation of the pathway across tissues and pathological contexts | [50,51,52] |
| OCDO → 27H-OCDO† | Established | Established | Established | Established: CYP27A1 | Supported | Established in experimental models: antiproliferative/antitumor activity and opposition to OCDO effects | Unresolved | Molecular target; quantitative in vivo flux; tissue-specific concentrations and contribution to endogenous regulation of OCDO signaling | [53] |

**Footnotes to Table S2**

Evidence categories. **Established** indicates that the corresponding level of evidence has been demonstrated experimentally and does not imply that the pathway is quantitatively dominant in all biological contexts. **Supported** denotes substantial experimental evidence for the indicated relationship while acknowledging that quantitative flux, tissue contribution, or pathway completeness remains incompletely resolved. **Partial/context-dependent** indicates evidence restricted to particular experimental systems or conditions. **Unresolved** indicates that the corresponding level of evidence has not yet been established. Evidence categories are evaluated independently; demonstration at one level does not imply demonstration at subsequent levels.

† Historical 27-hydroxy terminology is used as defined in Supplementary Table S1.
