# Supplementary material for: Defining the oxysterolome: analytical coverage, metabolic connectivity, and biological validation
Source: J Lipid Res. 2026 Oct 12;67(11):101170. doi: 10.1016/j.jlr.2026.101170 (PMC13761991; doi:10.1016/j.jlr.2026.101170)
Supplement: Supplementary file 3 [file mmc3.docx]

**Supplementary References**

**1.** Schenck GO, Neumüller OA, Eisfeld W. **Zur photosensibilisierten Autoxydation der Steroide: Δ5-Steroid-7α-hydroperoxyde und -7-ketone durch Allylumlagerung von Δ6-Steroid-5α-hydroperoxyden.** Justus Liebigs Ann Chem. 1958;618:202–210. doi:10.1002/jlac.19586180123.

**2.** Nedzvetskii SV, Paniukov AN, Shpats TA. **Стерины животного организма: 7-(α)-оксихолестерин.** Biokhimiia. 1953;18:315–318. PMID:13208719.

**3.** Russell DW. **The enzymes, regulation, and genetics of bile acid synthesis.** Annu Rev Biochem. 2003;72:137–174. doi:10.1146/annurev.biochem.72.121801.161712.

**4.** Ercoli A, de Ruggieri P. **The constitution of cerebrosterol, a hydroxycholesterol isolated from horse brain.** J Am Chem Soc. 1953;75:3284. doi:10.1021/ja01109a515.

**5.** Klyne W, Stokes WM. **The molecular rotations of polycyclic compounds. Part III. Polycyclic alcohols and their derivatives.** J Chem Soc. 1954:1979–1988.

**6.** Koizumi N, Morisaki M, Ikekawa N, Suzuki A, Takeshita T. **Absolute configurations of 24-hydroxycholesterol and related compounds.** Tetrahedron Lett. 1975;16:2203–2206. doi:10.1016/S0040-4039(00)72677-3.

**7.** Ercoli A, Di Frisco S, de Ruggieri P. **Isolamento, costituzione e significato biologico del cerebrosterolo, un compagno del colesterolo nel cervello di cavallo.** Boll Soc Ital Biol Sper. 1953;29:494–497. PMID:13105923.

**8.** Di Frisco S, de Ruggieri P, Ercoli A. **[Isolation of cerebrosterol from human brain].** Boll Soc Ital Biol Sper. 1953;29:1351–1352. PMID:13140512.

**9.** Lütjohann D, Breuer O, Ahlborg G, et al. **Cholesterol homeostasis in human brain: evidence for an age-dependent flux of 24S-hydroxycholesterol from the brain into the circulation.** Proc Natl Acad Sci USA. 1996;93:9799–9804. doi:10.1073/pnas.93.18.9799.

**10.** Björkhem I, Lütjohann D, Diczfalusy U, Ståhle L, Ahlborg G, Wahren J. **Cholesterol homeostasis in human brain: turnover of 24S-hydroxycholesterol and evidence for a cerebral origin of most of this oxysterol in the circulation.** J Lipid Res. 1998;39:1594–1600. PMID:9717719.

**11.** Ryer AI, Gebert WH, Murrill NM. **The preparation of cholesterol from 25-ketonorcholesterol.** J Am Chem Soc. 1950;72:4247–4248. doi:10.1021/ja01165a116.

**12.** Dauben WG, Bradlow HL. **The preparation of cholesterol from Δ5-norcholestene-3β-ol-25-one.** J Am Chem Soc. 1950;72:4248–4250. doi:10.1021/ja01165a117.

**13.** Fredrickson DS, Ono K. **The in vitro production of 25- and 26-hydroxycholesterol and their in vivo metabolism.** Biochim Biophys Acta. 1956;22:183–184. doi:10.1016/0006-3002(56)90236-0.

**14.** Lund EG, Kerr TA, Sakai J, Li WP, Russell DW. **cDNA cloning of mouse and human cholesterol 25-hydroxylases, polytopic membrane proteins that synthesize a potent oxysterol regulator of lipid metabolism.** J Biol Chem. 1998;273:34316–34327. doi:10.1074/jbc.273.51.34316.

**15.** Scheer I, Thompson MJ, Mosettig E. **5-Cholestene-3β,26-diol.** J Am Chem Soc. 1956;78:4733–4736. doi:10.1021/ja01599a054.

**16.** Fakheri RJ, Javitt NB. **27-Hydroxycholesterol, does it exist? On the nomenclature and stereochemistry of 26-hydroxylated sterols.** Steroids. 2012;77:575–577. doi:10.1016/j.steroids.2012.02.006.

**17.** Cali JJ, Russell DW. **Characterization of human sterol 27-hydroxylase. A mitochondrial cytochrome P-450 that catalyzes multiple oxidation reactions in bile acid biosynthesis.** J Biol Chem. 1991;266:7774–7778. PMID:1708392.

**18.** Mauthner J, Suida W. **Beiträge zur Kenntniss des Cholesterins.** Monatsh Chem. 1896. doi:10.1007/BF01519235.

**19.** Bergström S, Wintersteiner O. **Autoxidation of sterols in colloidal aqueous solutions. III. Quantitative studies on cholesterol.** J Biol Chem. 1942;145:309–319.

**20.** Hardegger E, Ruzicka L, Tagmann E. **Untersuchungen über Organextrakte. (3. Mitteilung). Zur Kenntnis der unverseifbaren Lipoide aus arteriosklerotischen Aorten.** Helv Chim Acta. 1943;26:2205–2221. doi:10.1002/hlca.19430260715.

**21.** Kantiengar NL, Morton RA. **Cholesta-3:5-dien-7-one in human atherosclerotic aortas.** Biochem J. 1955;60:25–28. doi:10.1042/bj0600025.

**22.** Brooks CJW, Harland WA, Steel G. **Squalene, 26-hydroxycholesterol and 7-ketocholesterol in human atheromatous plaques.** Biochim Biophys Acta. 1966;125:620–622. doi:10.1016/0005-2760(66)90055-5.

**23.** Brown AJ, Watts GF, Burnett JR, Dean RT, Jessup W. **Sterol 27-hydroxylase acts on 7-ketocholesterol in human atherosclerotic lesions and macrophages in culture.** J Biol Chem. 2000;275:27627–27633. doi:10.1074/jbc.M004060200.

**24.** Heo GY, Bederman I, Mast N, et al. **Conversion of 7-ketocholesterol to oxysterol metabolites by recombinant CYP27A1 and retinal pigment epithelial cells.** J Lipid Res. 2011;52:1117–1127. doi:10.1194/jlr.M014217.

**25.** Westphalen T. **Über die Einwirkung von Benzopersäure auf Cholesterin.** Ber Dtsch Chem Ges. 1915;48:1064–1069. doi:10.1002/cber.191504801149.

**26.** Plattner PA, Lang W. **Über die Konfiguration der beiden Cholesterin-oxyde und des “trans”-Cholestantriols-(3β,5,6).** Helv Chim Acta. 1944;27:1872–1880. doi:10.1002/hlca.194402701238.

**27.** Paillasse MR, Saffon N, Gornitzka H, Silvente-Poirot S, Poirot M, de Medina P. **Surprising unreactivity of cholesterol-5,6-epoxides towards nucleophiles.** J Lipid Res. 2012;53:718–725. doi:10.1194/jlr.M023689.

**28.** Gray MF, Lawrie TDV, Brooks CJW. **Isolation and identification of cholesterol α-oxide and other minor sterols in human serum.** Lipids. 1971;6:836–843. doi:10.1007/BF02531214.

**29.** Watabe T, Sawahata T. **Biotransformation of cholesterol to cholestane-3β,5α,6β-triol via cholesterol α-epoxide (5α,6α-epoxycholestan-3β-ol) in bovine adrenal cortex.** J Biol Chem. 1979;254:3854–3860. PMID:35533.

**30.** Aringer L, Eneroth P. **Formation and metabolism in vitro of 5,6-epoxides of cholesterol and β-sitosterol.** J Lipid Res. 1974;15:389–398. PMID:4152888.

**31.** de Medina P, Paillasse MR, Segala G, Poirot M, Silvente-Poirot S. **Identification and pharmacological characterization of cholesterol-5,6-epoxide hydrolase as a target for tamoxifen and AEBS ligands.** Proc Natl Acad Sci USA. 2010;107:13520–13525. doi:10.1073/pnas.1002922107.

**32.** Hattori J. **On the Rupture of Cholesterol Oxide. II.** Yakugaku Zasshi. 1940;60(6):334–339. doi:10.1248/yakushi1881.60.6_334.

**33.** Baxter RM, Spring FS. **Studies in the sterol group. Part XLVIII. Cholesterol β-oxide.** J Chem Soc. 1943:613–615. doi:10.1039/JR9430000613.

**34.** Nagano H, Poyser JP, Cheng KP, Luu B, Ourisson G. **Chemistry and biochemistry of Chinese drugs. Part II. Hydroxylated sterols, cytotoxic towards cancerous cells: synthesis and testing.** J Chem Res (S). 1977:218; J Chem Res (M). 1977:2522–2571.

**35.** Li D, Spencer TA. **Synthesis of 7α-hydroxy derivatives of regulatory oxysterols.** Steroids. 2000;65:529–535.

**36.** Toll A, Wikvall K, Sudjana-Sugiaman E, Kondo KH, Björkhem I. **7α-Hydroxylation of 25-hydroxycholesterol in liver microsomes. Evidence that the enzyme involved is different from cholesterol 7α-hydroxylase.** Eur J Biochem. 1994;224:309–316. doi:10.1111/j.1432-1033.1994.00309.x.

**37.** Schwarz M, Lund EG, Lathe R, Björkhem I, Russell DW. **Identification and characterization of a mouse oxysterol 7α-hydroxylase cDNA.** J Biol Chem. 1997;272:23995–24001. doi:10.1074/jbc.272.38.23995.

**38.** Ren S, Hylemon P, Zhang ZP, et al. **Identification of a novel sulfonated oxysterol, 5-cholesten-3β,25-diol 3-sulfonate, in hepatocyte nuclei and mitochondria.** J Lipid Res. 2006;47:1081–1090. doi:10.1194/jlr.M600019-JLR200.

**39.** Li X, Pandak WM, Erickson SK, et al. **Biosynthesis of the regulatory oxysterol, 5-cholesten-3β,25-diol 3-sulfate, in hepatocytes.** J Lipid Res. 2007;48:2587–2596. doi:10.1194/jlr.M700301-JLR200.

**40.** Pickard RH, Yates J. **Contributions to the chemistry of the cholesterol group. Part I. The action of hydrogen peroxide and of fused potassium hydroxide on cholesterol.** J Chem Soc Trans. 1908;93:1678–1687. doi:10.1039/CT9089301678.

**41.** Prelog V, Tagmann E. **Steroide und Sexualhormone. (105. Mitteilung). Über die Konfiguration des “cis”-Cholestan-triols-(3β,5,6).** Helv Chim Acta. 1944;27:1867–1871. doi:10.1002/hlca.194402701237.

**42.** Fioriti JA, Kanuk MJ, George M, Sims RJ. **Metabolic fate of epoxycholesterol in the rat.** Lipids. 1970;5:71–75. doi:10.1007/BF02531097.

**43.** de Medina P, Paillasse MR, Payré B, Silvente-Poirot S, Poirot M. **Synthesis of new alkylaminooxysterols with potent cell differentiating activities: identification of leads for the treatment of cancer and neurodegenerative diseases.** J Med Chem. 2009;52:7765–7777. doi:10.1021/jm901063e.

**44.** de Medina P, Paillasse MR, Segala G, et al. **Dendrogenin A arises from cholesterol and histamine metabolism and shows cell differentiation and anti-tumour properties.** Nat Commun. 2013;4:1840. doi:10.1038/ncomms2835.

**45.** Griffiths WJ, Gilmore I, Yutuc E, et al. **Identification of unusual oxysterols and bile acids with 7-oxo or 3β,5α,6β-trihydroxy functions in human plasma by charge-tagging mass spectrometry with multistage fragmentation.** J Lipid Res. 2018;59:1058–1070. doi:10.1194/jlr.D083246.

**46.** Beck KR, Inderbinen SG, Kanagaratnam S, et al. **11β-Hydroxysteroid dehydrogenases control access of 7β,27-dihydroxycholesterol to retinoid-related orphan receptor γ.** J Lipid Res. 2019;60:1535–1546. doi:10.1194/jlr.M092908.

**47.** Shoda J, Axelson M, Sjövall J. **Synthesis of potential C27-intermediates in bile acid biosynthesis and their deuterium-labeled analogs.** Steroids. 1993;58:119–125. doi:10.1016/0039-128X(93)90048-R.

**48.** Shoda J, Toll A, Axelson M, Pieper F, Wikvall K, Sjövall J. **Formation of 7α- and 7β-hydroxylated bile acid precursors from 27-hydroxycholesterol in human liver microsomes and mitochondria.** Hepatology. 1993;17:395–403. doi:10.1002/hep.1840170308.

**49.** Fieser LF, Rajagopalan S. **Selective oxidation with N-bromosuccinimide. II. Cholestane-3β,5α,6β-triol.** J Am Chem Soc. 1949;71:3938–3941. doi:10.1021/ja01180a016.

**50.** Roscoe HG, Fahrenbach MJ. **Metabolism of cholestane-3β,5α,6β-triol. II. Identification of two major neutral metabolites in the rat.** J Lipid Res. 1971;12:17–23. doi:10.1016/S0022-2275(20)39541-9.

**51.** Iuliano L, Crick PJ, Zerbinati C, et al. **Cholesterol metabolites exported from human brain.** Steroids. 2015;99:189–193. doi:10.1016/j.steroids.2015.01.026.

**52.** Voisin M, de Medina P, Mallinger A, et al. **Identification of a tumor-promoter cholesterol metabolite in human breast cancers acting through the glucocorticoid receptor.** Proc Natl Acad Sci USA. 2017;114:E9346–E9355. doi:10.1073/pnas.1707965114.

**53.** Ayadi S, et al. **27-Hydroxylation of oncosterone by CYP27A1 switches its activity from pro-tumor to anti-tumor.** J Lipid Res. 2023;64:100479. doi:10.1016/j.jlr.2023.100479.

**54.** Wang Y, Kumar N, Crumbley C, Griffin PR, Burris TP. **Modulation of retinoic acid receptor-related orphan receptor alpha and gamma activity by 7-oxygenated sterol ligands.** J Biol Chem. 2010;285:5013–5025. PMID:19965867.

**55.** Lund EG, Guileyardo JM, Russell DW. **cDNA cloning of cholesterol 24-hydroxylase, a mediator of cholesterol homeostasis in the brain.** Proc Natl Acad Sci USA. 1999;96:7238–7243.

**56.** Adams CM, Reitz J, De Brabander JK, Feramisco JD, Li L, Brown MS, Goldstein JL. **Cholesterol and 25-hydroxycholesterol inhibit activation of SREBPs by different mechanisms, both involving SCAP and Insigs.** J Biol Chem. 2004;279:52772–52780. doi:10.1074/jbc.M410302200.

**57.** Umetani M, Domoto H, Gormley AK, et al. **27-Hydroxycholesterol is an endogenous SERM that inhibits the cardiovascular effects of estrogen.** Nat Med. 2007;13:1185–1192. doi:10.1038/nm1641.

**58.** Nelson ER, Wardell SE, Jasper JS, et al. **27-Hydroxycholesterol links hypercholesterolemia and breast cancer pathophysiology.** Science. 2013;342:1094–1098. doi:10.1126/science.1241908.

**59.** Hannedouche S, Zhang J, Yi T, et al. **Oxysterols direct immune cell migration via EBI2.** Nature. 2011;475:524–527. doi:10.1038/nature10280.

**60.** Liu C, Yang XV, Wu J, et al. **Oxysterols direct B-cell migration through EBI2.** Nature. 2011;475:519–523. doi:10.1038/nature10226.

**61.** Ren S, Hylemon P, Zhang ZP, et al. **Sulfated oxysterol, 25HC3S, is a potent regulator of lipid metabolism in human hepatocytes.** Biochem Biophys Res Commun. 2007;360:802–808. doi:10.1016/j.bbrc.2007.06.143.

**62.** Zhang X, Bai Q, Xu L, et al. **Cholesterol metabolite, 5-cholesten-3β,25-diol-3-sulfate, promotes hepatic proliferation in mice.** J Steroid Biochem Mol Biol. 2012;132:262–270. doi:10.1016/j.jsbmb.2012.06.001.

**63.** Lin CY, Huo C, Kuo LK, et al. **Cholestane-3β,5α,6β-triol suppresses proliferation, migration, and invasion of human prostate cancer cells.** PLoS One. 2013;8:e65734. doi:10.1371/journal.pone.0065734.

**64.** Segala G, David M, de Medina P, et al. **Dendrogenin A drives LXR to trigger lethal autophagy in cancers.** Nat Commun. 2017;8:1903. doi:10.1038/s41467-017-01948-9.

**65.** Record M, Attia M, Carayon K, et al. **Targeting the liver X receptor with dendrogenin A differentiates tumour cells to secrete immunogenic exosome-enriched vesicles.** J Extracell Vesicles. 2022;11:e12211. doi:10.1002/jev2.12211.

**66.** Soroosh P, Wu J, Xue X, et al. **Oxysterols are agonist ligands of RORγt and drive Th17 cell differentiation.** Proc Natl Acad Sci USA. 2014;111:12163–12168. doi:10.1073/pnas.1322807111.
